# Supplementary material for: Relationship between circulating FSH levels and body composition and bone health in patients with prostate cancer who undergo androgen deprivation therapy: The BLADE study
Source: eLife. 2024 Apr 24;13:e92655. doi: 10.7554/eLife.92655 (PMC11042799; doi:10.7554/eLife.92655)
Supplement: Supplementary file 1. [file elife-92655-supp1.docx]

**SUPPLEMENTARY TABLE 1**

| Variable | | Study cohort  (29 patients) |
| --- | --- | --- |
| Age | Median (IQR) | 71 (63-79) |
| Smoking habits, n (%) | No | 19 (65.5) |
|  | Yes/Former | 10 (34.5) |
| CCI, n (%) | 0 | 18 (62.1) |
|  | ≥1 | 11 (37.9) |
| Diabetes, n (%) | No | 27 (93.1) |
|  | Yes | 2 (6.9) |
| Hypercholesterolemia, n (%) | No | 22 (75.9) |
|  | Yes | 7 (24.1) |
| Baseline PSA (ng/mL) | Median (IQR) | 3.7 (1.1-8.4) |
| GGG, n (%) | I-II | 7 (24.1) |
|  | III | 4 (13.8) |
|  | IV-V | 18 (62.1) |
| T stage, n (%) | T2 | 10 (34.5) |
|  | T3-T4 | 19 (65.5) |
| N stage, n (%) | N0 | 13 (44.8) |
|  | N1-N3 | 16 (55.2) |
| Primary treatment, n (%) | RP | 20 (69.0) |
|  | RT | 5 (17.2) |
|  | ADT | 4 (13.8) |
| Indication to enrollment, n (%) | Adjuvant after RP | 14 (48.3) |
|  | Salvage after RP or RT | 9 (31.0) |
|  | ADT with/without RT | 6 (20.7) |

Abbreviations: IQR = interquartile range; CCI = Charlson Comorbidity Index; PSA = prostate specific antigen; GGG = Gleason grade group; RP = radical prostatectomy, RT = radiation therapy; ADT = androgen deprivation therapy
